# Combinatorial pretreatment and fermentation optimization enabled a record yield on lignin bioconversion

**DOI:** 10.1186/s13068-018-1021-3

**Published:** 2018-01-29

**Authors:** Zhi-Hua Liu, Shangxian Xie, Furong Lin, Mingjie Jin, Joshua S. Yuan

**Affiliations:** 1 Synthetic and Systems Biology Innovation Hub (SSBiH), Texas A&M University, College Station, TX 77843 USA; 2 Department of Plant Pathology and Microbiology, Texas A&M University, College Station, TX 77843 USA; 3 Institute for Plant Genomics and Biotechnology, Texas A&M University, College Station, TX 77843 USA; 4 School of Environmental and Biological Engineering, Nanjing University of Science and Technology, Nanjing, 210094 China; 5 Guangdong Cleamol LTD, Foshan, 528225 China

**Keywords:** Lignin valorization, Lipid, Fed-batch fermentation, Combinatorial pretreatment, Detoxification, *Rhodococcus opacus* PD630

## Abstract

**Background:**

Lignin valorization has recently been considered to be an essential process for sustainable and cost-effective biorefineries. Lignin represents a potential new feedstock for value-added products. Oleaginous bacteria such as *Rhodococcus opacus* can produce intracellular lipids from biodegradation of aromatic substrates. These lipids can be used for biofuel production, which can potentially replace petroleum-derived chemicals. However, the low reactivity of lignin produced from pretreatment and the underdeveloped fermentation technology hindered lignin bioconversion to lipids. In this study, combinatorial pretreatment with an optimized fermentation strategy was evaluated to improve lignin valorization into lipids using *R. opacus* PD630.

**Results:**

As opposed to single pretreatment, combinatorial pretreatment produced a 12.8–75.6% higher lipid concentration in fermentation using lignin as the carbon source. Gas chromatography–mass spectrometry analysis showed that combinatorial pretreatment released more aromatic monomers, which could be more readily utilized by lignin-degrading strains. Three detoxification strategies were used to remove potential inhibitors produced from pretreatment. After heating detoxification of the lignin stream, the lipid concentration further increased by 2.9–9.7%. Different fermentation strategies were evaluated in scale-up lipid fermentation using a 2.0-l fermenter. With laccase treatment of the lignin stream produced from combinatorial pretreatment, the highest cell dry weight and lipid concentration were 10.1 and 1.83 g/l, respectively, in fed-batch fermentation, with a total soluble substrate concentration of 40 g/l. The improvement of the lipid fermentation performance may have resulted from lignin depolymerization by the combinatorial pretreatment and laccase treatment, reduced inhibition effects by fed-batch fermentation, adequate oxygen supply, and an accurate pH control in the fermenter.

**Conclusions:**

Overall, these results demonstrate that combinatorial pretreatment, together with fermentation optimization, favorably improves lipid production using lignin as the carbon source. Combinatorial pretreatment integrated with fed-batch fermentation was an effective strategy to improve the bioconversion of lignin into lipids, thus facilitating lignin valorization in biorefineries.

**Electronic supplementary material:**

The online version of this article (10.1186/s13068-018-1021-3) contains supplementary material, which is available to authorized users.

## Background

Biorefineries have been widely studied due to energy security, economic sustainability, and environmental concerns [1–4]. The economical biorefinery industry depends on the utilization of the complete cell wall (carbohydrates and lignin). Carbohydrates can be effectively converted into fermentable sugars to produce biofuels. However, conventional biorefineries, including those for cellulosic ethanol, along with the pulp and paper industries generate approximately 112 million tons of lignin “waste” annually in the United States alone [5, 6]. Such lignin is usually burned for heat and electricity with low-value utilization [2, 7–9]. Given the accessibility of lignin, the second most abundant natural polymer on earth after cellulose, lignin valorization will not only enable new uses for value-added products, but also be an essential process for the sustainable and competitive biorefinery industry.

However, the low reactivity of lignin in lignocellulosic biomass hinders its high-value utilization. Lignin, used by plants for structure composition, water transport, and defense, is a highly complex phenylpropanoid biopolymer that is derived from three aromatic monomers (*p*-coumaryl alcohol, coniferyl alcohol, and sinapyl alcohol) [10, 11]. Lignin monomers have various functional groups, such as methoxyl, carbonyl groups, phenolic hydroxyl, and aliphatic hydroxyl [12, 13]. These monomers are conjugated via various bonds, mainly including β–β, β-*O*-4-aryl ether linkages, and β-5 linkages. These properties reinforce the resistance of the lignin polymer to depolymerize using biological and chemical methods, which have hindered both its isolation and widespread application. Therefore, a promising strategy that combines effective fractionation with bioconversion technology should be developed to both facilitate and advance lignin valorization [6, 14–16].

**Fig. 1:**
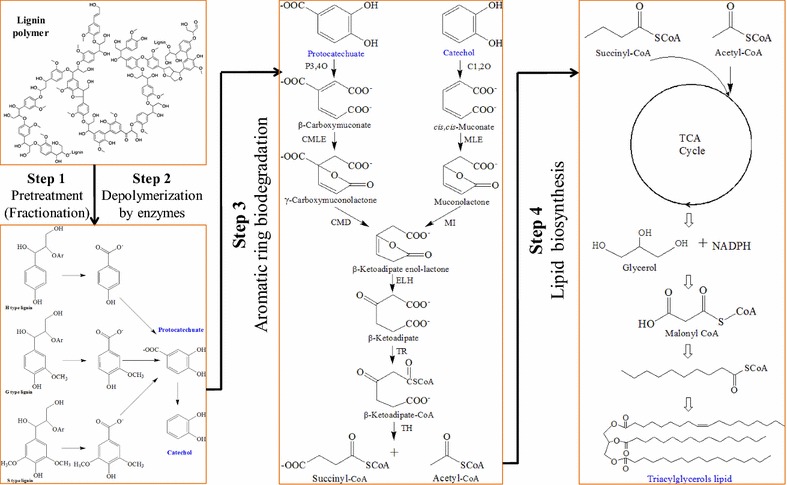
Major steps of lignin bioconversion from lignocellulosic biomass. Step 1, lignin fractionation; Step 2, lignin depolymerization by enzymes; Step 3, aromatic ring biodegradation in *Rhodococcus opacus*; Step 4, lipid synthesis in *Rhodococcus opacus*. P3, 4O is protocatechuate 3,4-dioxygenase; C1, 2O is catechol 1,2-dioxygenase; CMLE is β-carboxy-*cis,cis*-muconate lactonizing enzyme; MLE is *cis,cis*-muconate lactonizing enzyme; CMD is *γ*-carboxy-muconolactone decarboxylase; MI is muconolactone isomerase; ELH is β-ketoadipate enol-lactone hydrolase; TR is β-ketoadipate succinyl-CoA transferase; TH is β-ketoadipyl-CoA thiolase [7, 25, 47]

Oleaginous microorganisms can accumulate intracellular lipids to more than 20% of their cell dry weight [17, 18]. The fatty acids of those lipids are mainly long chain ones, which have been considered to be alternative fuel precursors for a more sustainable biodiesel industry [19–21]. Recently, lignin bioconversion into lipid by oleaginous microorganisms has attracted extensive attention due to their potential to add value and improve sustainability of biorefineries [6, 22–24]. Specifically, heterotrophic bacteria, such as *Rhodococcus opacus*, have evolved proficient catabolic networks (β-ketoadipate pathways) that are capable of degrading and converting lignin to produce lipids [25–27]. As shown in Fig. 1, similar to carbohydrate processing, there are four major steps of lignin bioconversion from lignocellulosic biomass: pretreatment, enzyme depolymerization, aromatic ring biodegradation, and lipid biosynthesis by microorganisms. In general, lignin polymers in lignocellulosic biomass are fractionated by pretreatment into low molecular weight lignin derivatives and then depolymerized into aromatic monomers by enzymes for subsequent bioconversion. However, two important factors will affect this process: (1) lignin–carbohydrate complexes hinder the lignin fractionation performance [28–31] and (2) the fractionation method alters the chemical bonds and functional groups of lignin, which determine the reactivity and bioconversion efficiency of lignin. However, the effects of different pretreatments on the bioconversion performance of lignin for lipid production are unclear.

For biodegradation and bioconversion of lignin, bacteria generally convert aromatic compounds into protocatechuate and catechol and then transfer them into β-ketoadipate, succinyl-CoA, and acetyl-CoA via β-ketoadipate pathways [12, 23, 25, 32]. In the case of oleaginous bacteria, succinyl-CoA and acetyl-CoA can be consumed to produce triacylglycerols from lipid biosynthetic pathways (Fig. 1). Under normal conditions, the product yield in most microorganisms is sensitive to a number of environmental factors [33, 34]. Lipid production by *Rhodococcus opacus* is no exception and is determined by the strain as well as the fermentation conditions. For example, the medium composition considerably affects the growth of strains and the production of lipids. To maximize lipid production, optimization of the medium composition such as carbon source (C), nitrogen source (N), and C/N ratio is of great importance. In general, increases in the lipid concentration are also dependent on the substrate concentration. Unfortunately, increases in the concentration of lignin derivatives will increase the concentration of lignocellulose-derived compounds generated from pretreatment. These compounds probably inhibit strain growth and thus reduce lipid accumulation in cells. Furthermore, an increased substrate concentration may also increase the viscosity of the medium, thus affecting strain growth and process handling. In addition to optimization of pretreatment, fed-batch fermentation and a detoxification strategy may provide ways to reduce inhibition and increase the cell biomass and lipid yield [35–37]. However, these strategies have not been optimized to improve lignin bioconversion into lipids. More importantly, there is no work showing how improvement of lignin reactivity via pretreatment optimization can be integrated with fermentation optimization to improve bioconversion. To overcome these challenges and allow for high-value utilization of lignin, effective fractionation that is integrated with fermentation optimization should be developed.

The aim of this work is to improve lignin bioconversion into lipids by developing a pretreatment process and fermentation strategy. The media compositions, especially the carbon and nitrogen sources, were optimized. The combinatorial pretreatment approach was used to fractionate lignin from corn stover. The effects of combinatorial pretreatment on lignin bioconversion were studied. Fermentation modes and detoxification strategies were constantly evaluated to remove the inhibition effects of derivatives on lipid fermentation. Furthermore, different fermentation modes were conducted in scale-up lipid fermentation in a 2.0-l fermenter. This process design and optimization is crucial to improve lipid production using lignin as a carbon source by *R. opacus* PD630.

## Methods

### Pretreatment strategies of corn stover

Lignin was fractionated from corn stover by pretreatment using a combination of dilute sulfuric acid, liquid hot water, sodium hydroxide, and ethanol (Table 1). Pretreatment was conducted following previous procedure [38]. The pretreatment conditions used in this study were the optimal conditions determined in our lab. During Step 1 of pretreatment, 50 g of corn stover (dry weight, dw) was loaded into a 1.0-l screw bottle at 10% (w/w) solid loading using dilute sulfuric acid or liquid hot water pretreatment heating by Amsco^®^ LG 250 Laboratory Steam Sterilizer (Steris, USA). The pretreated slurry from Step 1 was filtered by vacuum filtration to separate the solid fraction from the liquid stream. The solid fraction (loaded as described above) was then pretreated using sodium hydroxide and/or ethanol in Step 2. The liquid stream containing lignin was collected for lipid fermentation. The process using a combination of dilute sulfuric acid or liquid hot water in Step 1 with sodium hydroxide and/or ethanol in Step 2 was named combinatorial pretreatment [38].

**Table 1:** Combinatorial pretreatment strategies to fractionate lignin from corn stover [38]

Case	Step 1			Step 2		
	Chemicals	Conditions	Solid loading (%)	Chemicals	Conditions	Solid loading (%)
1	1% NaOH	120 °C, 60 min	10	–	–	–
2	Liquid hot water	120 °C, 30 min	10	1% NaOH	120 °C, 60 min	10
3	Liquid hot water	120 °C, 30 min	10	50% ethanol + 1% NaOH	120 °C, 60 min	10
4	1% H_2_SO_4_	120 °C, 30 min	10	1% NaOH	120 °C, 60 min	10
5	1% H_2_SO_4_	120 °C, 30 min	10	50% ethanol + 1% NaOH	120 °C, 60 min	10

% was calculated based on the weight percent, w/w

### Microorganism and seed culture preparation

*Rhodococcus opacus* PD630 was purchased from the Leibniz Institute DSMZ-German Collection of Microorganisms and Cell Cultures (Braunschweig, Germany). Engineered *R. opacus* PD630_FA was used in this study to grow on minimal medium was prepared as follows. First, 1.4 g of (NH_4_)_2_SO_4_ and 1.0 g of MgSO_4_·7H_2_O were added into 962 ml of ddH_2_O, and autoclave sterilized at 121 °C for 20 min. One milliliter 15 g/l of CaCl_2_·2H_2_O, 1.0 ml of a trace element solution, 1.0 ml of stock A solution, and 35.2 ml of 1.0 M phosphate buffer were then added to the solution and finalized to 1.0 l.

The trace element solution was composed of 0.5-g/l FeSO_4_·7H_2_O, 0.4-g/l ZnSO_4_·7H_2_O, 0.02-g/l MnSO_4_·H_2_O, 0.015-g H_3_BO_3_, 0.01-g/l NiCl_2_·6H_2_O, 0.25-g/l EDTA, 0.05-g/l CoCl_2_·6H_2_O, and 0.005-g/l CuCl_2_·2H_2_O. Stock A solution was composed of 2.0-g/l NaMoO_2_·2H_2_O and 5.0-g/l FeNa·EDTA.

A single colony of *R. opacus* PD630 was inoculated in 10 ml of Tryptic Soy Broth (TSB) medium at 28 °C for approximately 12–15 h. *R opacus* PD630 was then inoculated in 100 ml of secondary seed medium (TSB) at 28 °C with a shaking speed of 200 rpm for approximately 24 h to an OD_600_ 4.0.

### Lipid fermentation of the lignin stream from each pretreatment

Lipid fermentation, using lignin from each pretreatment as the carbon source, was carried out. The liquid stream containing lignin derivatives was adjusted to pH 7.0 by 1.0 M HCl and then sterilized at 121 °C for 15 min. For medium preparation, the liquid stream was dissolved to a particular soluble substrate concentration (SSC) by ddH_2_O and transferred into a 250-ml Erlenmeyer flask with a working volume of 100 ml. The medium consisted of 0.1 ml of 15-g/l CaCl_2_·2H_2_O, 0.1 ml of a trace-element solution, 0.1 ml of stock A solution, and 3.52 ml of 1.0 M phosphate buffer. *R. opacus* PD630 cell pellets, which were used for inoculation, were collected by centrifuging the seed culture at 5000 rpm for 10 min. Fermentation in 250-ml Erlenmeyer flasks was conducted at pH 7.0, 28 °C, and 200 rpm for 96 h. The scale-up fermentation was conducted in a 2.0-l fermenter with working volume of 1.0 l at pH 7.0, 28 °C, 60% pO_2_, and 200 rpm for 96 h. For fed-batch mode, lipid fermentation in cycle 1 was conducted at pH 7.0, 28 °C, and 200 rpm for 96 h. After cycle 1, the cell biomass was collected by centrifugation at 5000 rpm for 10 min and reused in cycle 2 by feeding a new lignin medium. Tables 2 and 3 show the lipid fermentation strategies in 250-ml Erlenmeyer flasks and the 2.0-l fermenter, respectively.

**Table 2:** Effects of inoculation OD, nitrogen source, soluble substrate, and fed-batch mode on the lipid fermentation of lignin stream by *Rhodococcus opacus* PD630

Parameters	Effects of OD	Effects of nitrogen source	Effects of soluble substrates	Effects of fermentation mode
Inoculum density (OD)	0.5, 1.0, 4.0, 8.0	1.0	1.0	1.0
(NH_4_)_2_SO_4_ (g/l)	1.4	0, 0.7,1.4, 2.1,2.8	1.4	1.4
Soluble substrate (g/l)	15	15	7.5, 15, 30, 45	45
Fermentation mode	Batch	Batch	Batch	Batch: 45 g/l (0 h)
				Fed-batch 1: 15 g/l (0 h) + 30 g/l (72 h)
				Fed-batch 2: 15 g/l (0 h) + 15 g/l (72 h) + 15 g/l (144 h)

Lignin stream used in the fermentation optimization was produced from NaOH pretreatment (Case 1, as shown in Table 1)

**Table 3:** Scale-up lipid fermentation strategies of lignin stream by engineered *Rhodococcus opacus* PD630_FA in a 2.0-l fermenter

Experiment no.	Substrate	Laccase treated	Fermentation mode	Initial OD	SSC (g/l)	Total fermentation time (h)	Nitrogen source (g/l)
1	Lignin 1	No	Batch	1.0	10 at 0 h	96	1.4
2	Lignin 1	No	Batch	10	40 at 0 h	96	1.4
3	Lignin 1	No	Fed-batch	10	20 at 0 h + 20 at 72 h	168	1.4
4	Lignin 4	No	Batch	1.0	10 at 0 h	96	1.4
5	Lignin 4	No	Fed-batch	5.0	20 at 0 h + 20 at 72 h	168	1.4
6	Lignin 4	Yes	Fed-batch	5.0	20 at 0 h + 20 at 72 h	168	1.4

Lignins 1 and 4 represent the lignin substrate fractionated by pretreatment Cases 1 and 4, as described in Table 1, respectively

*SSC* soluble substrate concentration

### Detoxification of lignin stream

To study the effects of detoxification on lipid fermentation, detoxification of the lignin stream using heating (H), activated carbon (AC), and gas stripping (G) was employed. For heating detoxification, the liquid stream was loaded into a 500-ml round-bottom reaction flask and heated in a water bath at 90 °C for 1 h. For activated carbon, 5% (w/v) activated carbon was added into the lignin stream in a 500-ml flask and shaken at 200 rpm for 12 h at room temperature. After detoxification, activated carbon was removed by vacuum filtration using a 0.2-μm filter membrane. For gas stripping, the lignin stream was added in a 500-ml flask, and the air was put into the bottom of the flask by a vessel with a distributor to perform stripping for 3 h.

### Laccase treatment of the lignin stream

To further depolymerize lignin, the lignin stream produced from the pretreatment was treated with laccase from trametes versicolor (Sigma-Aldrich, USA). Laccase treatment of lignin was conducted at a temperature of 50 °C and agitation of 200 rpm for 48 h in a 1.0 M phosphate buffer (pH 7.0) inside a 1-l flask with a breathable sealing film. 1-hydroxybenzotriazole hydrate (HBT) (Sigma-Aldrich, USA) was used as the mediator. Laccase loading of 15-mg/g substrate and a ratio 3:5 of laccase and HBT were used.

### Measurement of growth cell biomass and lipid concentration

After fermentation, 100-ml fermentation broth was centrifuged at 5000 rpm for 10 min. The cell pellets were washed in physiological salt solution and then freeze-dried by lyophilizer for 24 h. Cell dry weight (CDW) was recorded to trace cell growth. The supernatant was collected for analyzing soluble substrate concentration and lignin weight loss.

For lipid concentration analysis, the freeze-dried cell pellet was suspended with 20-ml menthol and mixed well. After incubating in 65 °C water bath for 30 min, 1.0-ml 10 N NaOH was added and the solution was incubated at 65 °C for 2 h. After that, 1.0-ml 98% (w/w) H_2_SO_4_ was slowly added, and the solution was then incubated in 65 °C water bath for another 2 h. The solution was cooled down to room temperature, and 8.0-ml hexane was added. The mixture was vigorously shaken for 5 min and then centrifuged at 4000 rpm for 10 min. The top hexane layer was transferred into a labeled and pre-weighted glass vial (weight recorded as *W*_1_). An additional 8-ml hexane was added and vigorously shaken for 5 min, and then centrifuged at 4000 rpm for 10 min. The top hexane layer was again transferred into the previous glass vials. The hexane glass vials were dried to constant weight (weight recorded as *W*_2_). The lipid content in cell biomass was calculated as follows:

1 $${\text{Lipid content}}\,\left( {{\text{g}}/{\text{g}}} \right) = \left( {W_{ 2} - W_{ 1} } \right)/M_{\text{cell dry weight}}$$

where *M*_cell dry weight_ is the weight of freeze-dried cell biomass.

### Derivative analysis by gas chromatography–mass spectrometry (GC–MS)

Lignin stream samples were centrifuged at 10,000*g* for 10 min. Supernatant was acidified to pH 0.5–1.0 with concentrated HCl. 3.0-ml acidified supernatant was mixed with 1.0-ml butanedioic acid-*d*_6_ as the internal standard, and then extracted with three volumes of methyl *tert*-butyl ether (MTBE) at 4500 rpm for 30 min. The organic layer was collected and dried under a stream of nitrogen gas. 5.0-ml MTBE was added to dissolve the sample. The sample was then filtered by 0.22-μm filter membrane for GC–MS analysis.

GC–MS was performed on GCMS-QP2010SE (Shimadzu Scientific Instruments, Inc.) using a Shimadzu SH-Rxi-5Sil column (30 m × 250 µm × 0.25 µm). An aliquot of 1 μl of the eluted sample was analyzed using helium as a carrier gas at a flow rate of 1.0 ml/min. The temperature profile of the GC method was 3 min at 50 °C, and then, it was increased to 290 °C at 8 °C/min. Ions were generated by a 70-eV electron beam at an ionization current of 40 μA. Mass spectral peak identification and quantification were performed using the GCMS solution software Ver. 2.6. The height of each acquired peak was normalized against that of internal standard for further data processing.

### Analysis methods

The sugars were analyzed by Ultimate 3000 HPLC System (Thermo Scientific, USA) equipped with an Aminex HPX-87P carbohydrate analysis column (Bio-Rad Laboratories, CA) and a refractive index detector using HPLC grade water as the mobile phase at a flow rate of 0.6 ml/min. SSC of the lignin stream was determined by the dry weight method using a 105 °C oven. Lignin concentration was analyzed following the Laboratory Analysis Protocol (LAP) of the National Renewable Energy Laboratory (NREL), Golden, CO, USA. Error bars in the tables and figures represented the standard deviation of the replicates. For all significance tests, a student *t* test was used requiring a probability *p* < 0.05 to be significant.

## Results and discussion

### Lipid fermentation optimization using lignin as the carbon source

**Fig. 2:**
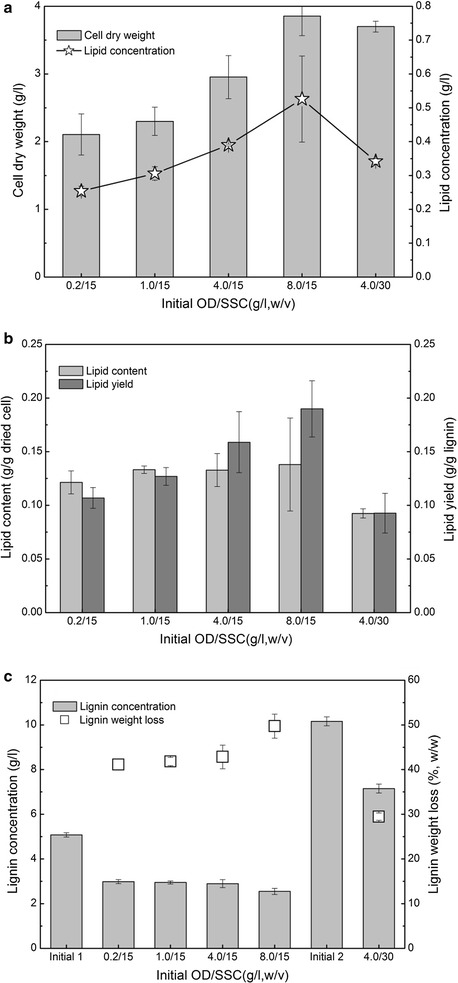
Effects of inoculum OD on the lipid fermentation performance of lignin by *R. opacus* PD630. **a** cell dry weight and lipid concentration. **b** lipid content and yield. **c** lignin concentration and weight loss. Fermentation conditions: 1.4-g/l (NH_4_)_2_SO_4_, pH 7.0, 30 °C, 200 rpm, and 96 h. SSC is soluble substrate concentration. *Initial 1* the initial lignin concentration at 15-g/l SSC, *Initial 2* the initial lignin concentration at 30-g/l SSC

**Fig. 3:**
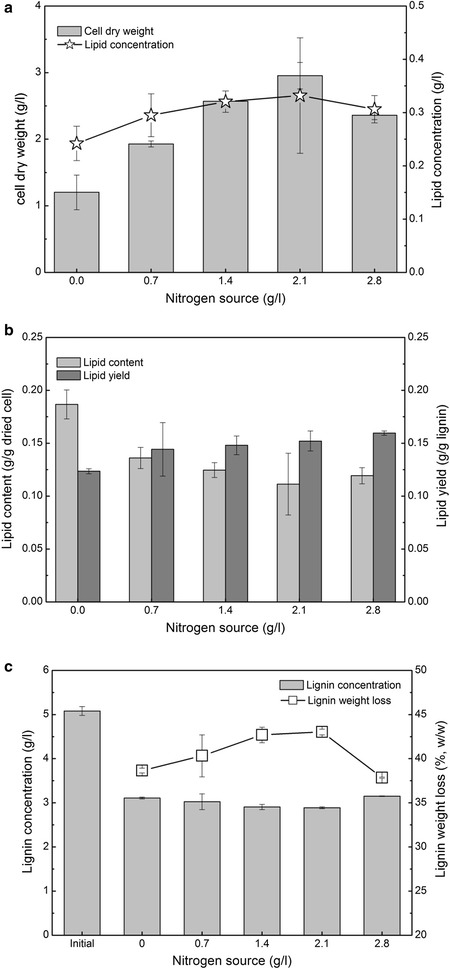
Effects of nitrogen source on the lipid fermentation performance of lignin by *R. opacus* PD630. **a** cell dry weight and lipid concentration. **b** lipid content and yield. **c** lignin concentration and weight loss. Fermentation conditions: 15-g/l SSC, OD 1.0, pH 7.0, 30 °C, 200 rpm, and 96 h. SSC is soluble substrate concentration. *Initial* the initial lignin concentration

Lignin valorization enhanced the overall biorefinery competitiveness, since lignin can be used as a potential feedstock to produce high-value products such as microbial lipids [6, 15]. Alkaline fractionated lignin (e.g., NaOH) is a potential carbon source that is utilized by Oleaginous *R. opacus* PD630 for lipid production due to its low molecular weight and high activity [15, 19]. In general, substrate consumption and the target product yield depend on the fermentation conditions. Thus, the effects of the fermentation parameters on lipid fermentation, including inoculum density (OD) and nitrogen source, were optimized (Figs. 2, 3).

Inoculum density is one of the most important factors that affects fermentation performance [39, 40]. A high inoculum density should certainly be conducive to initiating fermentation and increasing the product yield. However, a high inoculum density requires more substrates for the seed culture, which increases the capital cost of fermentation. The effects of the inoculum density on lipid fermentation of the lignin stream produced from NaOH pretreatment were recorded. As shown in Fig. 2a, the cell dry weight increased from 2.11 to 3.86 g/l when the inoculum OD increased from 0.2 to 8.0. These results suggested that a high inoculum OD led to a higher cell dry weight. One possible reason for these results is that the lignin stream produced from NaOH pretreatment contains various degraded products, such as acids, furans, and aromatic compounds, that are generated from carbohydrates, lignin, and other compositions in the lignocellulosic biomass [13, 35]. Some of these compounds may exhibit inhibition effects on *R. opacus* PD630. A high inoculum density should improve the tolerability of *R. opacus* PD630 to these inhibitors and thus increase the cell biomass. Another possible reason for these results is that a higher inoculum density itself should contribute to a higher cell biomass at the end of fermentation. Notably, the cell dry weight only increased by 25.4%, while the SSC increased from 15 to 30 g/l at OD4.0 (Fig. 2a). The lipid concentration increased from 0.25 to 0.53 g/l with the increase of the inoculum density. Unfortunately, the lipid concentration decreased by 12.8% when the SSC increased from 15 to 30 g/l at OD4.0. It is possible that SSC increases led to increases of the inhibitors, which in turn reduced the fermentable strain growth and thus lipid accumulation.

The lipid content increased from 0.12- to 0.14-g/g dried cell with the increase of the inoculum density, while the lipid yield increased from 0.10- to 0.19-g/g lignin (Fig. 2b). These results suggested that the high inoculum OD improved both the lipid content and lipid yield. A previous study reported that *Rhodococci* can convert ethanol organosolv lignin (EOL) and ultrasonicated EOL to lipids and that the highest lipid content is 4.08% of the dried cell [41]. These results demonstrated that NaOH fractionated lignin is a suitable substrate for lipid production. Compared with that at 15-g/l SSC, the lipid content and yield were only 0.09-g/g dried cell and 0.093-g/g lignin at 30-g/l SSC, respectively, which decreased by 30.4 and 41.6%. These results implied that the high SSC reduced the lipid content and lipid yield. The lignin concentration, at the end of fermentation, decreased with the increase of the inoculum density (Fig. 2c). The lignin weight loss was 41–49% at 15-g/l SSC, while only 25% at 30-g/l SSC at OD4.0. The lignin consumption results supported the cell dry weight and lipid concentration findings. All of these results showed that a high inoculum density led to a high lipid fermentation performance. In summary, because of the high cost of the seed preparation due to using a high inoculum density, OD1.0 was used as the inoculum density for fermentation optimization.

Lipid accumulation in oleaginous microorganisms depends on the excess carbon source and a limited nitrogen source in the medium [42, 43]. The oleaginous potential was critically affected by the nitrogen source or carbon-to-nitrogen ratio [44–46]. The results showed that, similar to its effect on many other oleaginous species, the nitrogen concentration plays a key role in lipid accumulation of *R. opacus* PD630 (Fig. 3). Under 15-g/l SSC and OD1.0, the cell dry weight sharply increased from 1.2 to 2.95 g/l when the nitrogen source increased from 0 to 2.1 g/l. The cell dry weight then decreased to 2.36 g/l with a 2.8-g/l nitrogen source (Fig. 3a). The lipid concentration increased from 0.24 to 0.33 g/l when the nitrogen source increased from 0 to 2.1 g/l. These results showed that nitrogen sources of 1.4 and 2.1 g/l almost produced the highest cell dry weight and lipid concentration. These results suggested that the nitrogen source should affect both the cell growth and lipid concentration. The previous work showed that *R. opacus* DSM 1069 and PD630 can convert the lignin model compounds into lipids under a C/N ratio of 5:1 to 10:1 [47]. In addition, a previous study also reported that both carbon and nitrogen sources have a significant effect on cell growth and microbial lipid accumulation [48]. Interestingly, the lipid content decreased from 0.19- to 0.11-g/l dried cell with the nitrogen source increases from 0 to 2.1 g/l, while the lipid yield increased from 0.12- to 0.16-g/l lignin (Fig. 3b). These results suggested that a high nitrogen source slightly increases the lipid yield, but it obviously decreases the lipid content in *R. opacus* PD630. An adequate nitrogen source is helpful for the growth of the cell biomass, but adverse to the accumulation of lipids. A high lipid content in the cell is helpful for improving the lipid extraction efficiency. The lignin weight loss was highest with a nitrogen source of 1.4 and 2.1 g/l. The lignin concentration and weight loss trends supported the above results. These results implied that *R. opacus* PD630 consumed more lignin substrate with a high nitrogen source to produce a higher cell-dried weight and lipid concentration. However, the high nitrogen source decreased the lipid content. Considering the nitrogen source usage, a 1.4-g/l nitrogen source was chosen as the optimal concentration.

### Enhancive lipid concentration with increased lignin concentration

**Fig. 4:**
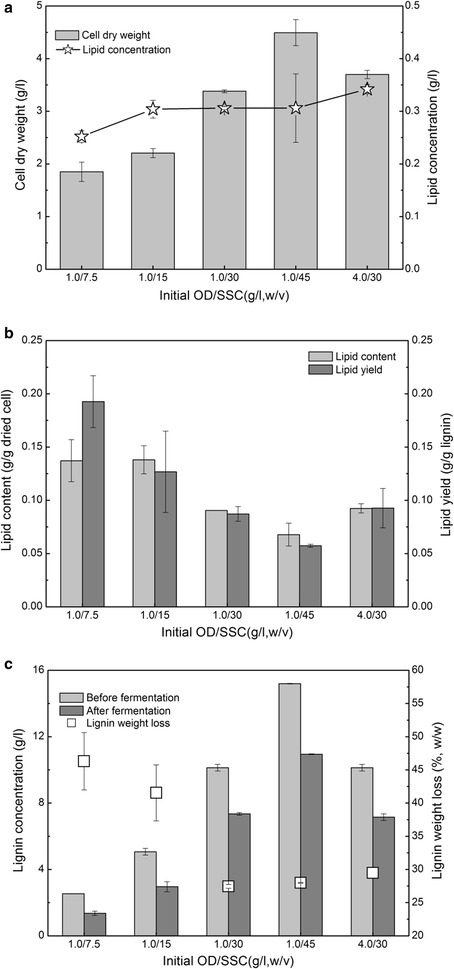
Effects of soluble substrate concentration (SSC) on the lipid fermentation performance of lignin by *R. opacus* PD630. **a** cell dry weight and lipid concentration. **b** lipid content and yield. **c** lignin concentration and weight loss. Fermentation conditions: 1.4-g/l (NH_4_)_2_SO_4_, pH 7.0, 30 °C, 200 rpm, and 96 h. *SSC* soluble substrate concentration

A high substrate concentration is usually needed to produce a high product concentration, which is helpful to reduce the separation cost. However, as mentioned, a high SSC in the medium contains high concentrations of degradation products and reduces strain growth due to the inhibition effect and rheology behavior change [49, 50]. The effects of the SSC on lipid production were investigated (Fig. 4). The results showed that with the SSC increasing from 7.5 to 45 g/l, the cell dry weight significantly increased from 1.9 to 4.5 g/l (Fig. 4a). The lipid concentration at 15-g/l SSC was 1.24 times higher than that at 7.5-g/l SSC; however, it hardly changed with a further increase of the SSC. These results suggested that a high SSC increased the cell dry weight, but hardly increased the lipid concentration. Interestingly, the cell dry weight and lipid concentration at OD 4.0 and 30-g/l SSC increased by 14.2 and 9.7%, respectively, compared to those at OD1.0. These results implied that a high inoculum density can increase the lipid concentration at a high SSC.

Figure 4b shows that the lipid content decreased from 0.14- to 0.07-g/g dried cell with the SSC increasing from 7.5 to 45 g/l, while the lipid yield decreased from 0.19- to 0.06-g/g lignin. The results implied that a high SSC produced a low lipid content and yield. Interestingly, the lipid content at OD4.0 was approximate to that with OD1.0 at 30-g/l SSC, but the lipid yield with OD4.0 was 6.3% higher than that with OD1.0. Results suggested that a high inoculum density produced a high lipid yield at a high SSC. Figure 4c shows that the lignin weight loss decreased from 46.3 to 27.9% with the SSC increasing from 7.5 to 45 g/l, which supported the results of the cell dry weight, lipid concentration, and lipid yield. Overall, the high SSC increased the cell dry weight and lipid concentration, but produced a low lipid content and lipid yield. There were no explanations for this phenomenon in the previous studies. The possible reason for this phenomenon was that a high SSC contains high concentrations of water extractives, ash, degradation products, and polymer segments generated from carbohydrates and lignin during pretreatment. These compounds may reduce the growth of *R. opacus* due to physical absorption and chemical inhibition effects. In addition, medium with a high SSC contains high salt concentrations that were introduced during pretreatment, which also decrease the lipid fermentation performance [13, 51]. To facilitate lipid production at a high SSC, effective strategies that eliminate these effects should be adopted.

### Improved lipid concentration by fed-batch fermentation

**Fig. 5:**
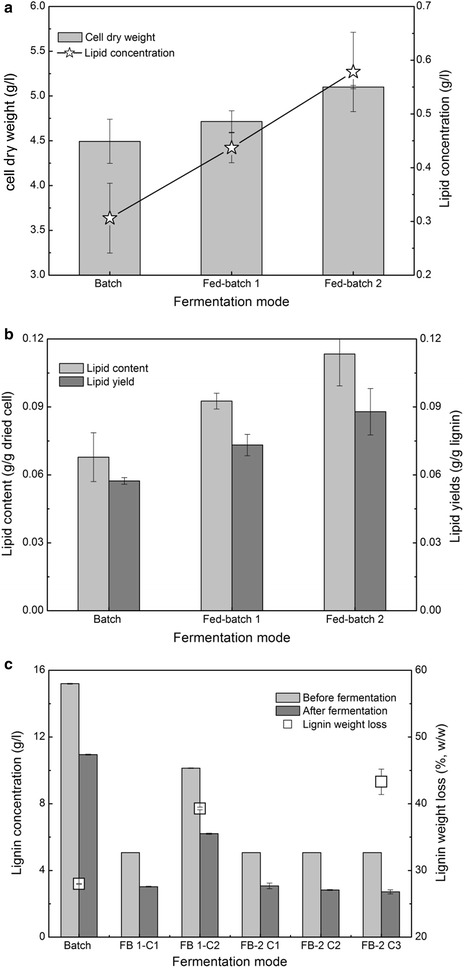
Effects of fed-batch mode on the lipid fermentation performance of lignin by *R. opacus* PD630. **a** cell dry weight and lipid concentration. **b** lipid content and yield. **c** lignin concentration and weight loss. Fermentation conditions: OD1.0, 1.4-g/l (NH_4_)_2_SO_4_, pH 7.0, 30 °C, and 200 rpm. *FB 1-C1* fed-batch mode 1 cycle 1, *FB 2-C1* fed-batch mode 2 cycle 1

Fed-batch fermentation is an operational technique in biotechnological processes, and can significantly reduce the inhibition effects of substrates and increase the cell concentration. The batch and fed-batch fermentation strategies by *R. opacus* PD630 were compared at a total SSC of 45 g/l (Table 2). Figure 5a shows that the cell dry weight in fed-batch fermentation modes 1 and 2 was 5.0 and 13.5% higher than those in the batch fermentation mode, respectively. The lipid concentrations in fed-batch fermentation modes 1 and 2 were 0.44 and 0.58 g/l, respectively, which were 1.42 and 1.9 times than those in the batch fermentation mode. Fed-batch fermentation, especially mode 2, increased the cell dry weight and lipid concentration at a high SSC compared to the batch fermentation mode.

Figure 5b shows that the lipid contents in the fed-batch fermentation modes 1 and 2 were 1.4 and 1.7 times those in the batch fermentation mode, respectively, while the lipid yields were 1.3 and 1.5 times those in the batch fermentation mode. These results showed that fed-batch fermentation mode 2 noticeably increased the lipid content and yield at a high SSC compared to the batch fermentation mode. Figure 5c shows that the lignin weight loss was only 28% for the batch fermentation mode. However, it reached 39.3 and 43.6% for fed-batch fermentation modes 1 and 2, respectively. These results prove that the fed-batch fermentation mode facilitates lipid production at a high SSC. It was deduced that the fed-batch fermentation mode may weaken the inhibitory effects by reducing the degradation product concentration, thus facilitating lipid fermentation.

### Combinatorial pretreatment improving lipid production from lignin

Lignin reactivity is closely related to various linkages and functional groups, which determine the lignin-based product yield in the bioconversion process. Fractionation technologies can significantly modify the lignin structure as well as change lignin reactivity and thus determine the lignin bioconversion performance. To improve the fractionation and bioconversion efficiency of lignin, combinatorial pretreatment was developed (Table 1). Combinatorial pretreatment with a low holding temperature has been confirmed to be a potential technology to maximize the output of fermentable sugars and lignin as well as improve lignin reactivity [38]. It avoids the disadvantages of each single pretreatment by reducing sugar degradation, inhibitor generation, and the need for energy consumption.

**Fig. 6:**
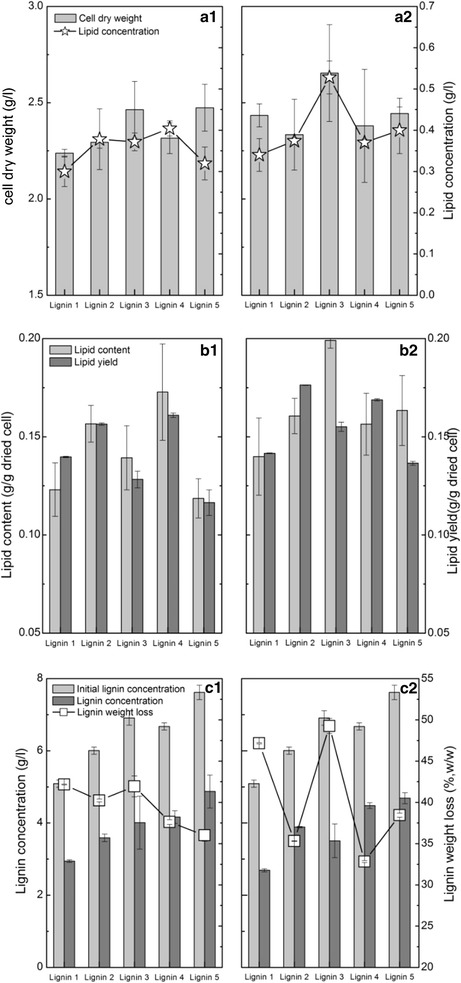
Lipid fermentation performance of lignin produced by combinatorial pretreatment using *R. opacus* PD630 (**a1**, **b1**, and **c1**) and engineered *R. opacus* PD630_FA (**a2**, **b2**, **c2**). Fermentation conditions: 15-g/l soluble substrate concentration, OD 1.0, 1.4-g/l (NH_4_)_2_SO_4_, pH 7.0, 30 °C, 200 rpm, and 96 h. *Lignin 1* lignin sample produced from pretreatment Case 1, as described in Table 1

To evaluate the lignin reactivity from each pretreatment, lignin (lignins 1–5 as labeled by its corresponding pretreatment Cases: 1–5) was used as the carbon source to produce lipids (Fig. 6). Compared to that from lignin 1, the cell dry weight from lignins 2–5 increased by 2.5 to 10.6% using *R. opacus* PD630 (Fig. 6a1). Using engineered *R. opacus* PD630_FA, the cell dry weight from lignins 2, 4, and 5 was approximated compared to that from lignin 1, but the cell dry weight from lignin 3 was 9.1% higher than that from lignin 1 (Fig. 6a2). Lignins 2–5 increased the lipid concentration by 6.5–34.8% compared to lignin 1. Lignin 4 produced the highest lipid concentration (0.4 g/l) among these lignin substrates (Fig. 6a1). The lipid concentration produced from lignins 2–5 using engineered *R. opacus* PD630_FA increased by 8.8–55.3% compared with that produced from lignin 1. Lignin 3 produced the highest lignin concentration (0.53 g/l) (Fig. 6a2). All of these results implied that lignins 2–5, produced from combinatorial pretreatment, noticeably increased the lipid concentration.

Figure 6b1 shows that the lipid content from lignins 2–5 using *R. opacus* PD630 was 13.2–40.3% higher than that from lignin 1. The highest lignin content was 0.17-g/l dried cell produced from lignin 4. Using engineered *R. opacus* PD630_FA, the lipid content in dried cell produced from lignins 2–5 was 11.8–42.3% higher than that from lignin 1. The highest lignin content was 0.20-g/l dried cell produced from lignin 3 (Fig. 6b2). Notably, engineered *R. opacus* PD630_FA had a 2.5–42.9% higher lipid content produced from lignins 1–5 than produce by *R. opacus* PD630. These results suggested that engineered *R. opacus* PD630_FA cells accumulated more lipid. The lipid yield produced from lignins 2 and 4 using *R. opacus* PD630 was 12.0–15.2% higher than that produced from lignin 1. The highest lipid yield (0.16 g/g lignin) was for that produced from lignin 4 (Fig. 61). Using engineered *R. opacus* PD630_FA, the lipid yield from lignins 2–5 was 9.5–24.5% higher than that from lignin 1, while the highest lipid yield (0.17-g/l lignin) was that produced from lignin 3 (Fig. 6b2). Notably, the lipid yield from lignins 4–5 using engineered *R. opacus* PD630_FA was 4.8–20.9% higher than that produced using *R. opacus* PD630. Lignin consumption during lipid fermentation was determined (Fig. 6c). The lignin weight loss was 36.0–42.2% for lignins 1–5 using *R. opacus* PD630, while it was 32.8–49.3% using engineered *R. opacus* PD630_FA. The glucose concentration in the lignin stream produced from each pretreatment was less than 2.3 g/l, which is much lower than the lignin concentration (Additional file 1). After fermentation, approximately 0.2–1.2-g/l glucose was consumed in lipid fermentation. The results also showed that the lignin and glucose in the liquid stream of the pretreatment were slightly different among these pretreatments, probably due to the difference in the pretreatment conditions [13, 51]. All of these results supported the results of the cell dry weight and lipid yield. All of the aforementioned results highlighted that the lipid production significantly depends on the types and reactivity of the lignin substrates. Lignin produced from combinatorial pretreatment, especially Cases 3 and 4, resulted in a higher lipid concentration, content, and yield compared to pretreatment Case 1 and thus improved the lipid fermentation performance.

**Fig. 7:**
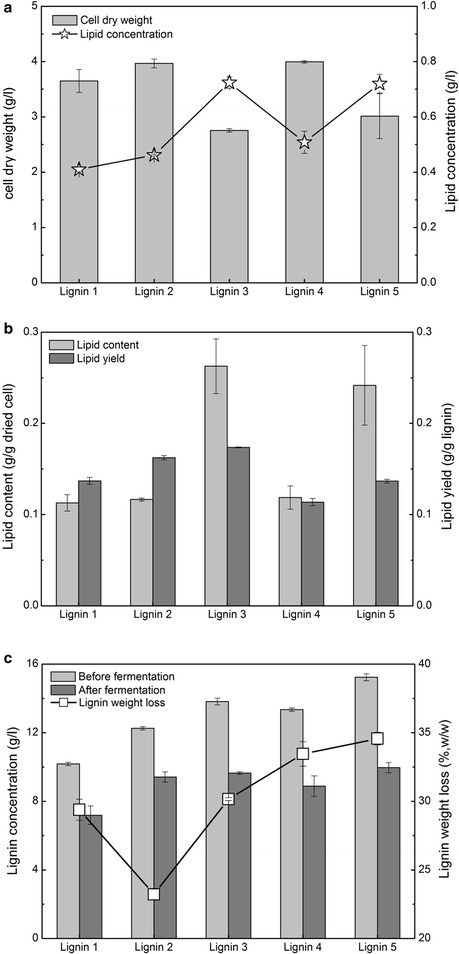
Lipid fermentation performance at high lignin concentration produced from combinatorial pretreatment by engineered *R. opacus* PD630_FA. **a** cell dry weight and lipid concentration. **b** lipid content and yield. **c** lignin concentration and weight loss. Fermentation conditions: 30-g/l soluble substrate concentration, OD 1.0, 1.4-g/l (NH_4_)_2_SO_4_, pH 7.0, 30 °C, 200 rpm, and 96 h. *Lignin 1* lignin sample produced from pretreatment Case 1, as described in Table 1

To increase the lipid concentration, fermentation was conducted at a high SSC (30 g/l) using engineered *R. opacus* PD630_FA (Fig. 7). Compared to that produced from lignin 1, the cell dry weight produced from lignins 2 and 4 increased by 8.7 and 9.5%, respectively, while it decreased by 24.4 and 17.2% from lignins 3 and 5 (Fig. 7a). However, the lipid concentration produced from lignins 2, 3, 4, and 5 was 12.8, 76.6, 23.9, and 75.6% higher than that produced from lignin 1, respectively. The highest lipid concentration (0.72 g/l) was produced from lignins 3 and 5. Compared to that at 15-g/l SSC, the lipid concentration at 30-g/l SSC increased by 20.6–80.0% from lignins 1–5. These results suggested that fermentation at a high SSC produced a high lipid concentration. The lipid contents in dried cell from lignins 2 and 4 were 3.3 and 5.2%, respectively, which were higher than that from lignin 1. Interestingly, the lipid contents were 0.26- and 0.24-g/g dried cell from lignins 3 and 5, respectively, which were 2.3 and 2.1 times greater than that from lignin 1. A possible reason for this result could be that during the conditioning of the lignin stream, rotary evaporation of the lignin stream in Cases 3 and 5 was conducted to recover ethanol. Volatile inhibitors may be concomitantly removed, which should facilitate lipid fermentation at a high SSC. The lipid yield from lignins 2 and 3 increased by 18.4 and 26.7% compared to that from lignin 1. From lignins 4 and 5, the lipid yield was approximated equivalent to that from lignin 1. Lignin weight loss from lignins 3, 4, and 5 was higher than that from lignin 1 at 30-g/l SSC, which supported the above lipid fermentation results well. All of the aforementioned results showed how lignins 2 and 4 increased the cell dry weight compared to lignin 1. Lignins 3 and 5 obviously increased the lipid concentration and content at a high SSC. These results showed that the lipid fermentation performance significantly depended on the reactivity of lignin produced from different fractionation methods.

**Fig. 8:**
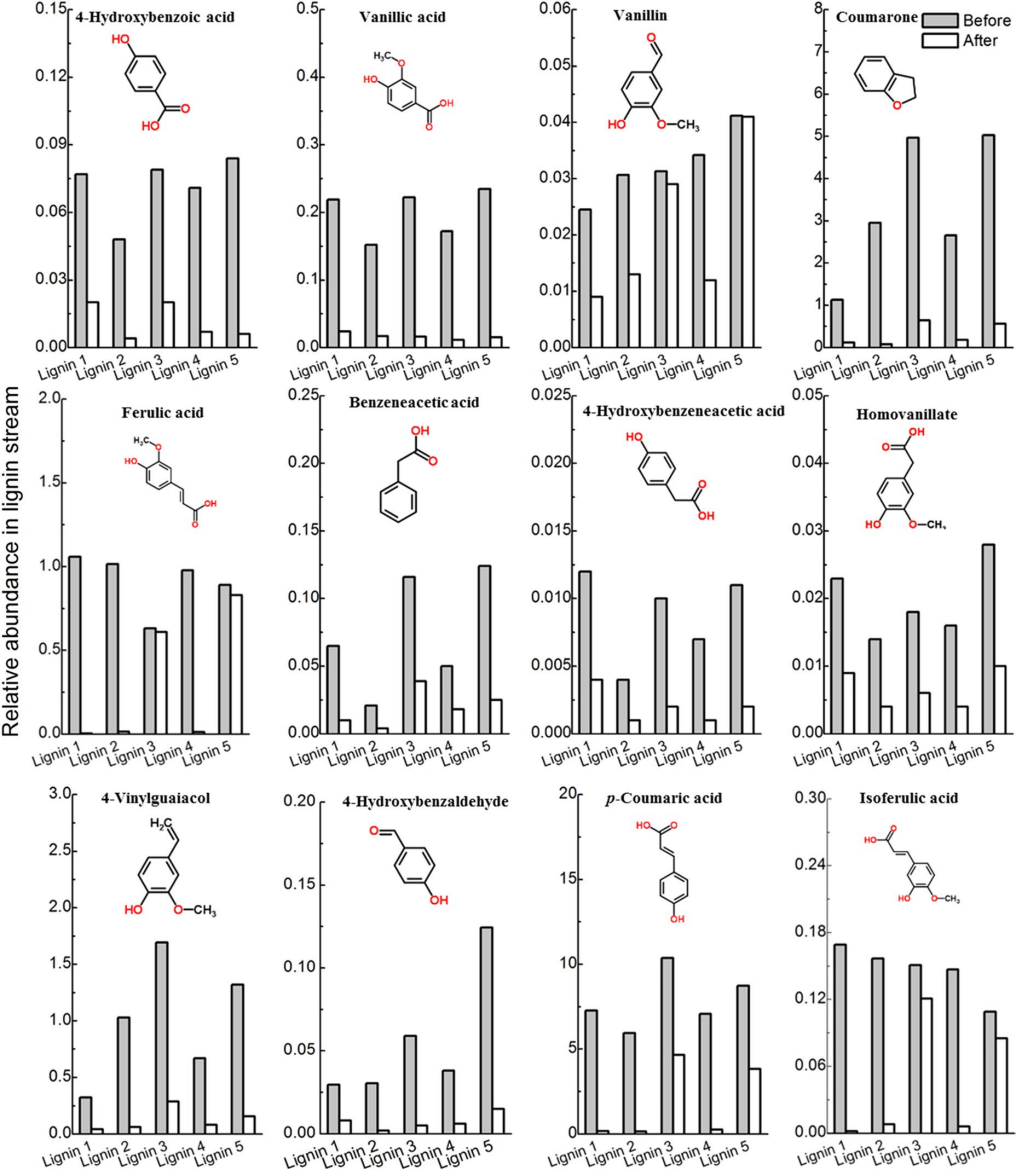
Relative abundance of major aromatic monomers in lignin stream before and after lipid fermentation by engineered *Rhodococcus opacus* PD630_FA. Fermentation conditions: 30-g/l soluble substrate concentration, OD 1.0, 1.4-g/l (NH_4_)_2_SO_4_, pH 7.0, 30 °C, 200 rpm, and 96 h. *Lignin 1* lignin sample produced from pretreatment Case 1, as described in Table 1

Aromatic monomers in the lignin medium prepared from each pretreatment were analyzed using GC–MS (Fig. 8). The results showed that the relative abundance of the aromatic monomers depended on the pretreatment options. In general, aromatic monomers are more easily utilized by lignin-degradable microorganisms, especially compared with a lignin polymer. Combinatorial pretreatment Cases 2–5 produced more aromatic monomers than pretreatment Case 1 and thus improved lignin bioconversion. Compared with that using NaOH (Cases 2 and 4) in Step 2, pretreatment using ethanol/NaOH (Cases 3 and 5) produced higher relative abundances of aromatic monomers, which should be helpful in increasing lipid fermentation. This result was consistent with the lipid fermentation results. By the end of lipid fermentation, the aromatic monomers, including 4-hydroxybenzoic acid, vanillic acid, coumarone, benzeneacetic acid, 4-hydroxybenzeneacetic acid, 4-vinylguaiacol, and *p*-coumaric acid, almost disappeared. These aromatic monomers contained a COOH group and were derived from *p*-coumaryl alcohol (H) and coniferyl alcohol (G) units. H- or G-type lignins were more likely to be consumed by lignin-degradable bacteria [47, 51]. A previous study also showed that the major aromatic compounds, such as vanillin, 2,3-dihydro-benzofuran, and 2,3-dimethoxybenzoic acid, disappeared after 168 h of fermentation by *R. opacus* PD630. Overall, compared to pretreatment Case 1, combinatorial pretreatment Cases 2–5 produced more aromatic monomers that were easily consumed, which contributed to the improvement of lipid fermentation.

### Detoxification of the lignin stream enhanced lipid fermentation at a high SSC

**Fig. 9:**
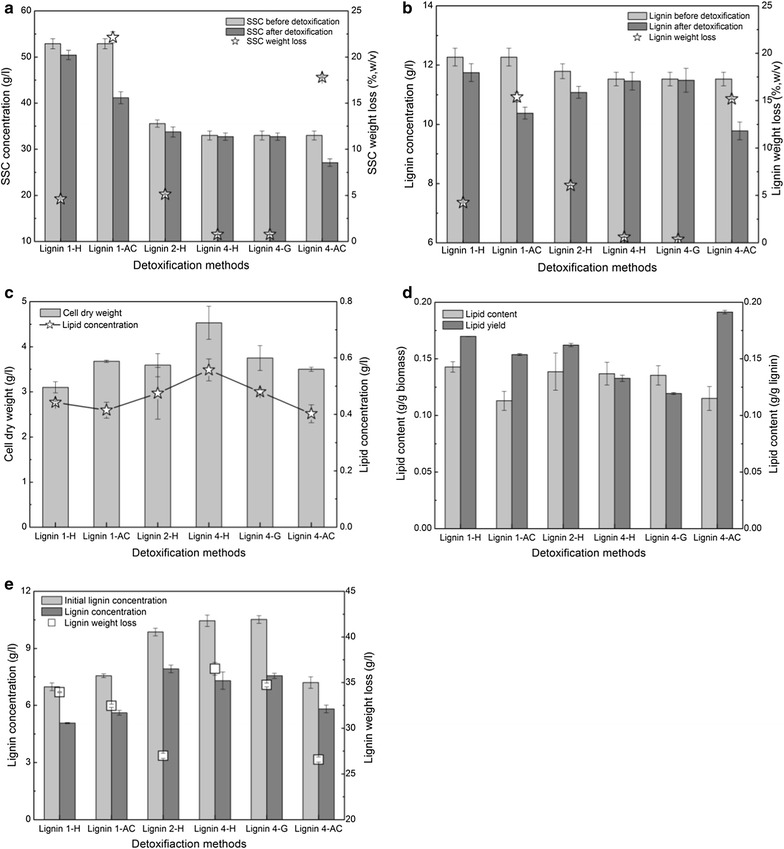
Lipid fermentation performance of lignin by engineered *R. opacus* PD630_FA with different detoxification methods. **a** SSC concentration and weight loss before and after detoxification. **b** lignin concentration and weight loss before and after detoxification; **c** cell dry weight and lipid concentration. **d** lipid content and yield. **e** lignin concentration and weight loss before and after fermentation. Fermentation conditions: 30-g/l soluble substrate concentration, OD 1.0, 1.4-g/l (NH_4_)_2_SO_4_, pH 7.0, 30 °C, 200 rpm, and 96 h. *Lignin 1-H* lignin 1 with heating detoxification, *Lignin 1-AC* lignin 1 with activated carbon detoxification, *lignin 4-G* lignin 4 with gas stripping detoxification, and *Lignin 1* lignin sample produced from pretreatment Case 1, as described in Table 1

Lignin streams produced from different pretreatments contain considerable amounts of degradation compounds. Previous reports have stated that degradation compounds have different inhibition effects on oleaginous strain growth and the product yield, which depend on the type of degradation compounds and oleaginous strains that are used [52, 53]. Before fermentation, detoxification of the liquid streams is required to remove the inhibitors that were formed in pretreatment. The effects of the detoxification methods, including heating (H), activated carbon (AC), and gas stripping (G), were evaluated (Fig. 9). Figure 9a, b shows that activated carbon detoxification removed 22.1 and 17.8% of the SSC for lignin 1-AC and lignin 4-AC and 15.4 and 15.2% of the lignin derivatives for lignin 1-AC and lignin 4-AC, respectively. These results are likely due to the physical adsorption effects of the soluble substrate and lignin derivatives on activated carbon. Heating detoxification removed 1.0–5.0% of the SSC and 1.0–6.0% of the lignin derivatives for lignins 1-H, 2-H, and 4-H, while gas stripping detoxification removed only 0.76% of the SSC and 0.38% of the lignin derivatives for lignin 4-G. These results suggested that the different detoxification strategies have various impacts on the SSC and lignin derivative concentration.

After detoxification, the cell dry weight produced from lignin 1-AC and lignin 4-H was higher than that without detoxification. The cell dry weight produced from lignin 1-H, lignin 2-H, lignin 4-G, and lignin 4-AC was lower than that without detoxification (Figs. 7a, 9c). The possible reason for these results, although detoxification removed most of the inhibitors, is that detoxification may have reduced the concentration of aromatic monomers and low molecular weight lignin derivatives, which are easily utilized. However, lignin 1-H, lignin 2-H, and lignin 4-H increased the lipid concentration by 7.3, 2.9, and 9.7%, respectively, compared to those without detoxification (Fig. 9c). Lignin 1-AC and lignin 4-G showed a similar lipid concentration to those without detoxification.

After detoxification, the lipid contents produced from lignin 1-H, lignin 2-H, lignin 4-H, and lignin 4-G increased by 21.0, 19.0, 15.4, and 14.2%, respectively, compared to those without detoxification. The lipid yields from lignin 1-H, lignin 1-AC, lignin 4-H, lignin 4-G, and lignin 4-AC increased by 19.3, 12.2, 16.8, 5.0, and 46.2%, respectively, compared to those without detoxification. The results suggested that detoxification of the lignin stream increased the lipid content and lipid yield. A possible reason for this increase is that detoxification may remove the inhibitors as well as facilitate strain growth and thus increase lipid accumulation in cells. All of these results highlighted that detoxification of the lignin stream was an effective strategy to improve the lipid fermentation performance.

### Scale-up lipid fermentation at a high soluble substrate concentration

**Fig. 10:**
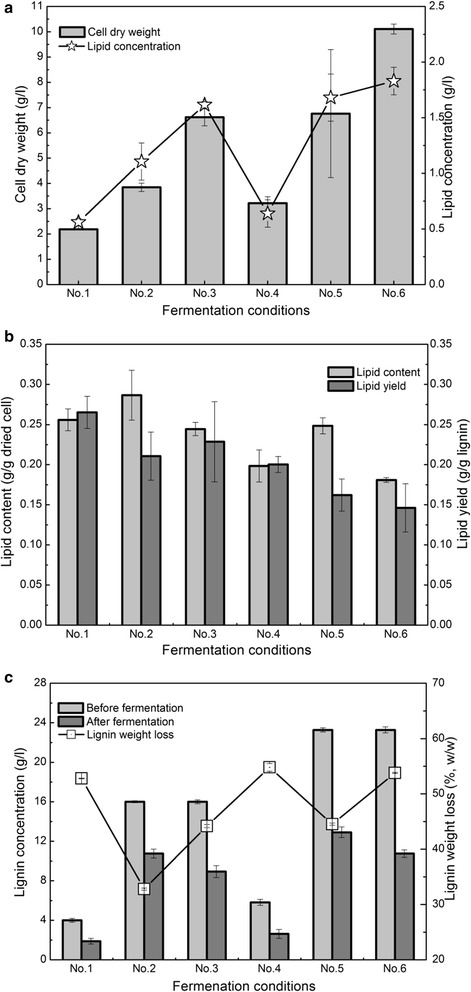
Lipid fermentation performance of lignin with batch or fed-batch fermentation using engineered *R. opacus* PD630_FA in 2.0-l fermenter. **a** cell dry weight and lipid concentration. **b** lipid content and yield. **c** lignin concentration and weight loss. Fermentation conditions: pH 7.0, 60% pO_2_, 30 °C, and 200 rpm. Nos. 1–6 represents the fermentation strategies, as shown in Table 3

Scale-up lipid fermentation, using lignin as a carbon source, was carried out in a 2.0-l fermenter (Table 3 and Fig. 10). Compared to that from lignin 1 at 10-g/l SSC with OD1.0 (No. 1 in Table 3), the cell dry weight and lipid concentration from lignin 4 increased by 47 and 14% (No. 4 in Table 3), respectively (Fig. 10a). The results suggested that combinatorial pretreatment Case 4 improved the scale-up lipid fermentation, which was consistent with the above results. To increase the lipid concentration, a total SSC of 40 g/l, with a high inoculation OD, was used in batch and fed-batch fermentation. As shown in Fig. 10a, although an inoculum OD10 was used (No. 2 in Table 3), the cell dry weight and lipid concentration from lignin 1 at 40-g/l SSC were only 1.7 and 1.9 times greater than those at 10-g/l SSC with OD1.0 (No. 1 in Table 3). Fed-batch fermentation was conducted at a total SSC of 40 g/l with an inoculum OD10 (No. 3 in Table 3), from which the cell dry weight and lipid concentration were 1.7 and 1.5 times greater than those of batch fermentation (No. 2 in Table 3), respectively. These results indicated that fed-batch fermentation increased the lipid fermentation performance of lignin 1.

Using lignin 4 as a carbon source (No. 5 in Table 3), the cell dry weight and lipid concentration with an inoculum at OD5.0 were 1.8 and 1.5 times greater than those from lignin 1 at OD10 (No. 2 in Table 3), respectively. Although an inoculum at OD5.0 was used in fed-batch fermentation (No. 5 in Table 3), the cell dry weight and lipid concentration from lignin 4 were higher than those from lignin 1 (No. 3 in Table 3). Laccase treatment was then used to depolymerize lignin to improve the lipid fermentation performance. As shown in Fig. 10a (No. 6 in Table 3), the highest cell dry weight and lipid concentration were 10.1 and 1.83 g/l, respectively, which were produced from lignin 4 with laccase treatment. The cell dry weight and lipid concentration in No. 6 were 1.5 and 1.2 times greater than those in No. 3 and 1.4 and 1.1 times greater than those in No. 5, respectively. These results suggested that laccase treatment of the lignin stream obviously improved the lipid fermentation performance, which may be due to the decreased molecular weight of lignin and increased lignin reactivity. Zhao et al. also reported that *R. opacus* cell growth increased exponentially in response to the level of laccase treatment of lignin [27]. The results showed that the cell dry weight and lipid concentration in a fermenter were higher than those in a flask, because the fermenter can provide adequate oxygen and control the pH of the medium accurately. A previous study also reported that a fed-batch culture in a stirred-tank fermenter produced a higher cell biomass, lipid content, and lipid productivity rate than that in a shaking flask [48]. Fed-batch fermentation of lignin 4 (Nos. 5 and 6 in Table 3) led to a lower lipid content and yield compared to those of lignin 1. It should be noted that a higher inoculum OD was used in Nos. 2 and 3, thereby contributing to the increased lipid content and yield. Overall, these results highlighted that combinatorial pretreatment integrated with fed-batch fermentation increased the lipid fermentation performance during scale-up fermentation.

## Conclusions

Combinatorial pretreatment was developed to fractionate lignin from corn stover, improve lignin reactivity, and ultimately, enhance lipid production. Combinatorial-pretreated lignins 2–5 increased the lipid concentration by 12.8–75.6% at 30-g/l SSC compared to single-pretreated lignin 1. The highest cell dry weight and lipid concentration in the fermenter were 10.1 and 1.83 g/l, respectively, which were produced from combinatorial-pretreated lignin 4, followed by laccase treatment in fed-batch fermentation. These results demonstrated that the lipid fermentation performance using lignin as a carbon source can be favorably improved by a combinatorial pretreatment with optimization of varying fermentation conditions.

## Additional file

**Additional file 1.** Additional table and figure.

## Abbreviations

AC: activated carbon
C: carbon source
CDW: cell dry weight
EOL: ethanol organosolv lignin
GC–MS: gas chromatography–mass spectrometry
GS: gas stripping
H: heating
N: nitrogen source
SSC: soluble substrate concentration
TSB: Tryptic Soy Broth
