# Supplementary material for: Combinatorial pretreatment and fermentation optimization enabled a record yield on lignin bioconversion
Source: Biotechnol Biofuels. 2018 Jan 29;11:21. doi: 10.1186/s13068-018-1021-3 (PMC5787925; doi:10.1186/s13068-018-1021-3)

**Additional information A** Components in lignin stream before and after lipid fermentation. The results of this table respond to the fermentation results shown in Figure 6 (A2, B2, C2). Fermentation conditions: 15 g/l soluble substrate concentration, OD 1.0, 1.4 g/l (NH_4_)_2_SO_4_, pH 7.0, 30 °C, 200 rpm, and 96 h. Lignin 1 stands for the lignin sample produced from pretreatment Case 1 as described in Table 1.

|  | Before fermentation | | | Before fermentation | | |  |
| --- | --- | --- | --- | --- | --- | --- | --- |
| Components/g/l | Lignin | Glucose | Others | Lignin | Glucose | Others | Ref. |
| Lignin 1 | 5.1 | 1.2 | 8.7 | 2.9 | 1.0 | - |  |
| Lignin 2 | 6.0 | 1.4 | 7.6 | 3.6 | 1.2 | - |  |
| Lignin 3 | 6.9 | 2.0 | 6.1 | 4.0 | 1.0 | - |  |
| Lignin 4 | 6.7 | 1.7 | 6.6 | 4.2 | 0.9 | - |  |
| Lignin 5 | 7.6 | 2.3 | 5.1 | 4.9 | 1.1 | - |  |
| Alkaline pretreated liquor (APL)* | 4.8 | 0.9 | 9.3 |  |  |  | [[13](#_ENREF_13)] |
| Alkali-extracted lignin (AEL) | 7.9 | 3 | 4.1 |  |  |  | [[51](#_ENREF_51)] |

* The components of alkaline pretreated liquor (APL) reported in previous study was based on the weight percent, w/w. Other components in lignin stream may include extractives, ash, salts, proteins, etc.

**Additional information B** The GC-MS data of major aromatic monomers in lignin stream 1 before and after lipid fermentation. Fermentation conditions: 30 g/l soluble substrate concentration, OD 1.0, 1.4 g/l (NH_4_)_2_SO_4_, pH 7.0, 30°C, 200 rpm, and 96 h. Lignin 1 stands for the lignin sample produced from pretreatment Case 1 as described in Table 1.


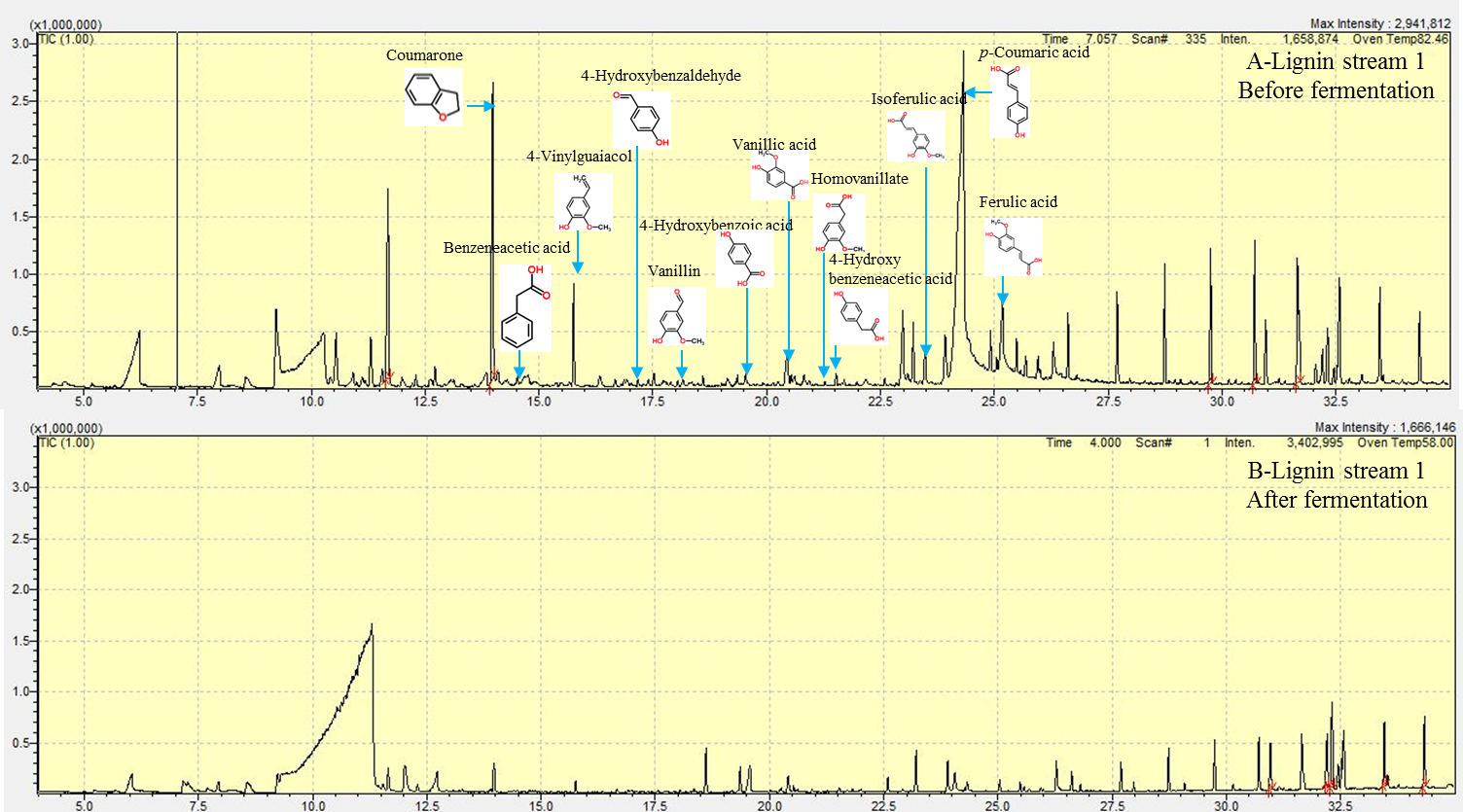

Supplement: Supplementary file 1 — Additional file 1. Additional table and figure. [file 13068_2018_1021_MOESM1_ESM.docx]
